# Melano-Sarcoma of the Female Urethra-Urethrectomy—Recovery1Read at the ninth annual meeting of the American Association of Obstetricians and Gynecologists at Richmond, Va., September 22-24,1896.

**Published:** 1896-12

**Authors:** Charles A. L. Reed

**Affiliations:** Gynecologist to the Cincinnati Hospital, St. Leger Place


### MELANO-SARCOMA OF THE FEMALE URETHRA—URETHRECTOMY—RECOVERY.[1]

By CHARLES A. L. REED, A. M., M. D.,
Gynecologist to the Cincinnati Hospital.

THERE is, I am sure, no occasion to apologise for bringing to the attention of the association the details of a case of melano-sarcoma of the female urethra ; a case which, so far as a reasonably comprehensive research of the literature indicates, is the first of the kind to be placed upon record. Sarcoma of even the ordinary varieties, involving the urethra, are exceedingly rare, there being, according to Ehrenderfer,[2] but two upon record—namely, one by Biegel,[3] in 1875, the other by Ehrenderfer himself, in 1892.[4] In neither of these cases, however, were melanotic features the distinguishing characteristics. I mention this fact not to emphasize my priority in either the observation or publication of this condition—a matter of no essential importance—but to indicate the extreme varity of the condition manifested in the following case :

Mary E. Y., *aet.* 64 ; single, was brought to my private hospital by her family physician, Dr. Joseph Morris, of Columbus Grove, Ohio, December 3, 1895. The patient had had no serious previous illness. There was no history of tuberculosis or syphilis in the family. The virginal condition of the genitalia precluded the supposition of venereal

---

1. Read at the ninth annual meeting of the American Association of Obstetricians and Gynecologists at Richmond, Va., September 22–24, 1896.

infection of any character. Her general health was good, although there was some emaciation about the neck and breasts, the latter of which were flabby, changes no doubt incident to age. Careful examination revealed no diseased conditions about either the lungs or heart. Careful palpation and percussion of the abdomen yielded negative results. There was no complaint of disturbance of either the gastric, renal or other functions. The urine was normal in color and quantity and had a specific gravity of 1.014. About eight months previously, *i. e.*, in April, 1895, she began to notice some pain, accompanied with blood on micturition. This was shortly followed by a more or less constant pinkish discharge from the genital fissure. The self-examination which followed revealed a tumor at the meatus urethræ. This tumor continued to increase in both size and hemorrhagic tendency until she was prompted to consult Dr. Morris, who curetted the neoplasm thoroughly and treated it with styptics. This brought apparent recovery, which endured but for only a short time, when the mass reappeared with granulations of greater exuberance than previously. The patient, however, declined to accept further operative interference until she was brought to me. At that time I found a black, lobulated and eroded mass, about 3 c.m. in diameter, separating the labiæ majora. The orifice of the urethra was in the very center of the mass. A careful vaginal examination was not made at the time, as the vaginal structures, present in their integrity, rendered such an exploration very painful.

The operation was done the next day, December 4th. The small blade of a Jones's speculum—an excellent self-retaining perineal retractor—was introduced. The patient being in the Simons' posture, the urethra was by this means exposed in its entire length. A longitudinal incision was made through the mucous membrane, along the dorsum of the urethra from a point where the presenting part of the mass was eroded to the base of the bladder. Another incision through the mucous membrane was made at right angles to the foregoing at a point far enough above the eroded mass to insure healthy tissue. The mucous membrane was then dissected back in two lateral flaps and the urethra was enucleated.

The urethra was found to be distinctly conical in shape, the base of the cone being at the meatus, the apex at the bladder. Care was taken to dissect out the canal to a point manifestly above the zone of malignant involvement. When this point was reached, but a slight distance from the bladder, the canal with its neoplastic walls was excised. The cut margin of the cystic segment of the canal was seized at various points in its circumference by Kocher's forceps, brought down by gentle traction and fixed by interrupted sutures of silkworm gut to the vaginal mucous membrane. A self-retaining catheter was inserted and the patient was put to bed. The sutures were removed on the eighth day. The catheter was dispensed with on the twelfth day. The patient sat up on the fourteenth day, when she found that she could retain her urine and void it at will. She was dismissed December 21st entirely healed.

She remained in good health until the 1st of July following—seven months—when she again summoned Dr. Morris because of some stomach symptoms. He found her suffering from persistent vomiting and with a large mass in the epigastrium. This mass rapidly increased in size until it occupied all of the area between the navel and the breast bone, its nodular characteristics becoming more and more pronounced. She died of exhaustion July 14, 1896, having had no recurrence whatever of the urethral trouble. No autopsy was permitted.

The specimen removed was a dark mass, tri-lobar and conical in shape, being about 2 c.m. in diameter at its base and about 25 m.m. in diameter at its apex. Its longitudinal diameter was approximately 3.5 c.m. It had a distinct investment capsule of loose connective tissue, which was broken at the margin of the eroded base. The base of the tumor was pierced in its center by the meatus urethræ, was eroded, black and presented the appearance of a diffuse ecchymosis. This dark coloring was found on sections of the tumor, after hardening, to depend upon diffuse pigmentation, which, however, was more marked in one lobe than in the remaining two. The cells are of the small round variety, although in some parts they are of a larger size. The stroma is very slight, while in some parts it is practically absent. Some pigmented cells may be found interspersed throughout the growth, while in one lobe the nonpigment cells are gathered in clusters, each cluster being surrounded by heavy deposits of pigment, most of which is found in cell bodies, but much of it is apparently enmeshed in the slender fibrillæ of the stroma. A cross-section of both the urethra and of Skene's follicles reveals the epithelium of the mucous membrane of each practically undisturbed. The investment capsule can be distinctly traced and consists of connective tissue, the inner surface of which alone presents slight evidence of round-cell invasion.

St. Leger Place.

